# Relationship Between Vitamin D Deficiency and Non-alcoholic Fatty Liver Disease: A Cross-Sectional Study From a Tertiary Care Center in Northern India

**DOI:** 10.7759/cureus.34921

**Published:** 2023-02-13

**Authors:** Manoj Kumar, Ashwin Parchani, Ravi Kant, Arindam Das

**Affiliations:** 1 Department of General Medicine, Uttar Pradesh University of Medical Sciences, Saifai, IND; 2 Department of General Medicine, All India Institute of Medical Sciences, Rishikesh, Rishikesh, IND

**Keywords:** non-alcoholic steatohepatitis, non-alcoholic fatty liver disease, insulin resistance, body mass index, vitamin d

## Abstract

Background

Vitamin D levels are strongly associated with myocardial infarction, coronary artery disease, heart dysfunction, and even mortality. Non-alcoholic fatty liver disease (NAFLD) is a prevalent hepatic illness whose incidence has grown dramatically over the past several decades.

Methodology

This observational, cross-sectional study was conducted over 1.5 years (January 2019 to June 2020) at the Department of General Medicine of a tertiary care hospital in northern India on 100 adult patients with NAFLD admitted to the emergency ward, intensive care unit, and medical ward.

Results

In our study, of the 100 patients, 45.0%, 16.0%, and 39.0% of patients exhibited vitamin D deficiency, insufficiency, and sufficiency, respectively. Vitamin D deficiency was the highest among those aged 41-50 (54.2%) and lowest among those aged 30-40 (29.0%). We observed that vitamin D deficiency was less prevalent in people with a normal body mass index (39.1%) than in those who were overweight (91.7%). There was a significant (p < 0.05) association between the severity of vitamin D deficiency and the presence of hepatomegaly, splenomegaly, and ascites. Overall, the incidence of fatty liver was 49% among patients. There was a significant (p = 0.0001) correlation between fatty liver and serum vitamin D levels. The association between the proportion of patients with fatty liver and the degree of vitamin D deficiency was found to be significant (p = 0.04). The relationship between the distribution of patients according to insulin resistance and the degree of vitamin D deficiency was also statistically significant (p < 0.001).

Conclusions

Vitamin D deficiency is associated with an increased risk of NAFLD, as well as with the severity of NAFLD.

## Introduction

The primary function of vitamin D is to control bone metabolism; nevertheless, its deficiency is associated with many other organ systems. Diabetes, hypertension, hyperlipidemia, and peripheral vascular disease are more prevalent in those with vitamin D deficiency. Vitamin D levels are also strongly linked to coronary artery disease (CAD), myocardial infarction, heart failure, stroke, and incident mortality [1]. Vitamin D deficiency, defined by low serum 25(OH)D levels, may develop from inadequate sun exposure, poor vitamin D consumption, or malabsorption. Vitamin D insufficiency is common among adults and ranges between 24% and 49% of the global population [2]. It has been shown to be associated with a variety of health issues. It is commonly recognized that vitamin D deficiency leads to osteoporosis, osteomalacia, and increased fracture risk.

Vitamin D deficiency has been previously linked to an increase in the prevalence of hypertension, diabetes, peripheral vascular disease, and hyperlipidemia [3]. Some studies also indicate that vitamin D levels are associated with obesity, inflammation, and insulin resistance [4]. In addition, it has been demonstrated that vitamin D supplementation lowers free fatty acid (FFA)-induced insulin resistance in animal models [5].

Non-alcoholic fatty liver disease (NAFLD) is perhaps the most common form of liver disease in adults with a worldwide prevalence of 32.4% [6]. Its prevalence has increased rapidly over the past few decades. NAFLD is an umbrella term that encompasses benign adipose tissue accumulation in the liver, progressive steatosis with hepatitis, fibrosis, cirrhosis, and, in rare instances, hepatocellular carcinoma (HCC). NAFLD comprises the following two conditions: non-alcoholic fatty liver (NAFL) and non-alcoholic steatohepatitis (NASH). NAFL is defined by liver steatosis involving more than 5% of the parenchyma without any signs of hepatocyte damage. Histologic criteria identify NASH as a necro-inflammatory process in which steatosis causes liver cells to become destroyed. Despite the fact that the natural history of NAFLD is still being investigated, there is a risk of progression to cirrhosis and HCC, according to the existing evidence. It is currently one of the most prevalent chronic liver diseases worldwide. According to its name, the primary hallmark of NAFLD is an abundance of fat deposits in liver cells. NASH is an aggressive form of fatty liver disease marked by liver inflammation that, in some NAFLD patients, can progress to cirrhosis and ultimately liver failure. This damage is comparable to that caused by excessive alcohol intake. NAFLD is described as the accumulation of fat in the liver in the absence of other factors such as viral hepatitis, alcohol abuse, and others. NAFLD is presently recognized as a significant component of metabolic syndrome [7] and has emerged as a developing clinical entity.

Numerous related pathways contribute to the development of NAFLD, and several potential risk factors, including metabolic syndrome, insulin resistance, and obesity, have been discovered. Several studies have shown that vitamin D levels in adults are inversely linked with NAFLD [8,9]. Vitamin D deficiency has long been considered a risk factor for the development of NAFLD. However, the association between vitamin D deficiency and NAFLD has only been investigated and assessed in a small number of studies.

## Materials and methods

Study setting and oversight

This observational, cross-sectional study was conducted in the Department of General Medicine at a tertiary care hospital in northern India on 100 adult patients with NAFLD admitted to the emergency ward, intensive care unit (ICU), and medical ward over the course of 1.5 years (January 2019 to June 2020). Patients who gave informed consent and fulfilled the inclusion criteria were enrolled in this research. The Institutional Ethics Committee of the Uttar Pradesh University of Medical Sciences granted ethical approval (reference number: 139/2018).

Patients

All adult (over 18 years of age) patients with newly diagnosed NAFLD were eligible for inclusion. Key exclusion criteria included patients with significant alcohol consumption or alcohol intake >20 g/day; patients with chronic liver disease, including those seropositive for hepatitis B and C viruses; patients exposed to metals such as antimony, barium salts, chromates, phosphorus, thallium compounds, and uranium compounds; patients who had undergone surgical procedures such as biliopancreatic diversion, extensive small bowel resection, gastric bypass and jejunoileal bypass, abnormal thyroid function test; and patients who refused to give consent.

Methodology

Patients who fulfilled the study’s inclusion criteria were identified. Patients were informed of the purpose of the research, and those who agreed to participate were enrolled in the study. After obtaining informed consent, each patient’s complete medical history was collected and recorded using a standardized questionnaire that covered demographics, leisure activities, past medical history, family medical history, medication history, nicotine and alcohol use, and dietary patterns. All measurements, including body height and weight, hip, and waist-to-hip ratio (WHR), were measured in accordance with the World Health Organization (WHO) recommendations. After eliminating alcohol consumption and viral or other liver illnesses, NAFLD was diagnosed using abdominal ultrasound findings and graded according to severity. A structured form was utilized to record the results of the examination. During the examination, the liver, gallbladder, kidneys, and spleen were among the organs evaluated. The liver size, presence of localized lesions, and hepatic steatosis were assessed. According to previous research [10-12], hepatic steatosis was diagnosed based on predetermined criteria. The presence of comorbidities such as hepatomegaly, splenomegaly, and ascites was also noted.

Complete blood count (CBC), fasting lipid profile, serum total cholesterol (TC) (cholesterol esterase method), high-density lipoprotein (HDL-C) (cholesterol esterase method after precipitation with phosphotungstate method), low-density lipoprotein (LDL-C) (Fried Wald’s Formula: LDL - C = TC - HDL - C + TG/5), viral markers, fasting blood sugar, fasting serum insulin, liver function test, and kidney function test estimates were performed using the Selectra Pro XL fully automated clinical chemistry analyzer with reagent (ELITechGroup, Puteaux, France). Viral markers were determined using chemiluminescent immunoassays. The CBC was calculated using an automated hematological analyzer (Sysmex XS-1000i, Sysmex Corporation, Kobe, Japan). Serum 25(OH)D levels were measured using an Achitect 25-OH vitamin D test (Abbott Diagnostics, Lake Forest, IL, USA). The assay has a minimum limit of detection of 2.8 ng/mL and a maximum limit of 147.8 ng/mL. 25(OH)D levels were categorized as deficient (20 ng/mL), insufficient (20-29 ng/mL), and sufficient (30 ng/mL).

Statistical analysis

Microsoft Office Excel version 2010 was used in creating the database and producing graphs, while the data were analyzed using SPSS version 26 for Windows (IBM Corp., Armonk, NY, USA). Various tests of significance were employed for the analysis of data, as mentioned in the Results section. Categorical data were evaluated using the chi-square test. A p-value <0.05 was used as the level of significance.

## Results

**Figure 1:**
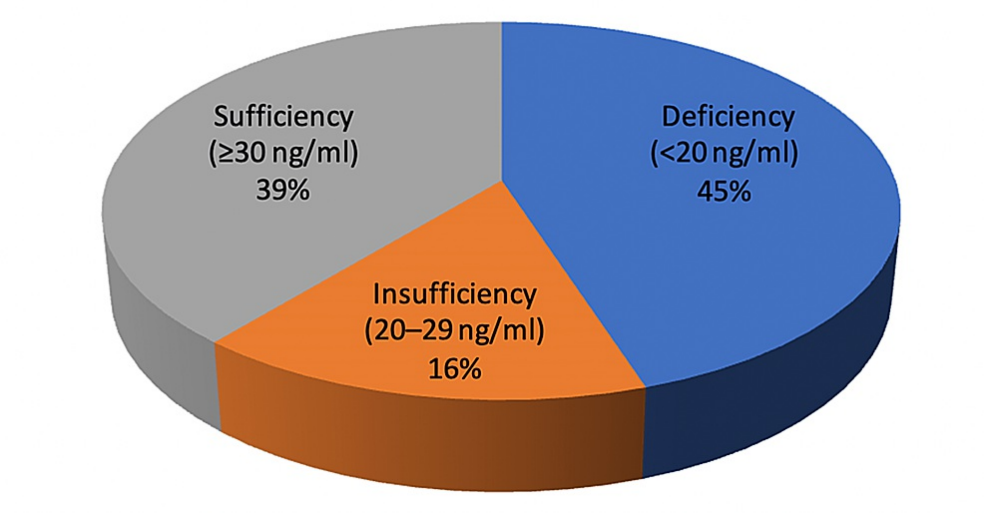
Serum vitamin D status in NAFLD patients. NAFLD = non-alcoholic fatty liver disease

**Figure 2:**
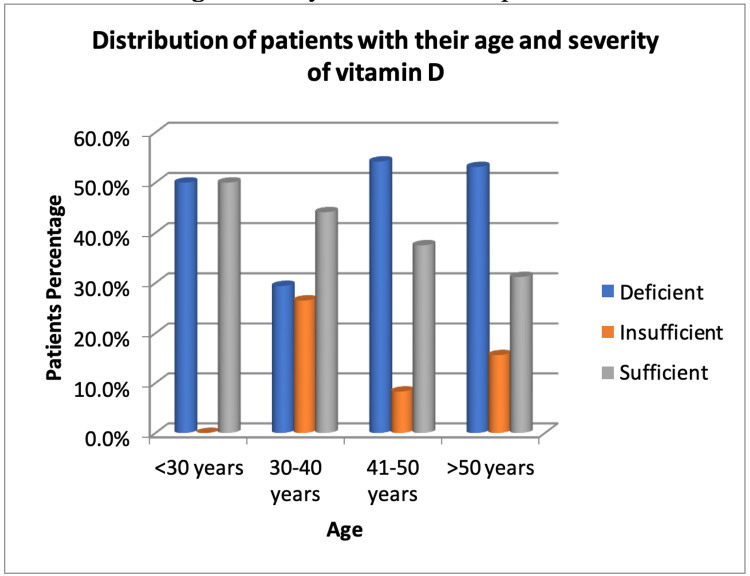
Distribution of patients based on age and serum vitamin D status.

In our study, of the total 100 patients, 45.0%, 16.0%, and 39.0% of patients were vitamin D deficient, insufficient, and sufficient, respectively (Figure 1). Overall, 34% of patients were between 30 and 40 years old, followed by 32% aged >50, 24% aged 41-50, and 10% aged 30 years. Vitamin D deficiency was the highest among those aged 41-50 (54.2%) and lowest among those aged 30-40 (29.0%) (Figure 2). However, there was no significant correlation between the severity of vitamin D insufficiency and age (p > 0.05). Further, 64% of the patients were female. Vitamin D deficiency was greater in females (46.9%) than in males (41.7%), although there was no significant correlation (p > 0.05) between the degree of vitamin D deficiency and gender.

The clinical history of patients is presented in Table 1, with abdominal discomfort being the most prevalent (18.0%) symptom. Not a single patient was an alcoholic. About 35% of patients were smokers. Vitamin D insufficiency was lower among smokers (42.9%) than among non-smokers (46.2%). The percentage of overweight (body mass index (BMI) ≥25) patients was 12%. Vitamin D deficiency was lower in normal people (39.1%) than in obese patients (91.7%). Overall, 9% and 21% of patients, respectively, exhibited splenomegaly and hepatomegaly. Vitamin D deficiency was greater among those with such comorbidities than among those without, with a significant (p < 0.05) correlation between severe vitamin D deficiency and the presence of comorbidity (Table 2). Table 3 depicts the relationship between several blood tests and the degree of vitamin D deficiency. All patients were negative for viral indicators. According to the degree of vitamin D deficiency, the results of the liver function test, lipid profile, uric acid, and platelet count were determined and investigated. The primary incidence of fatty liver in patients was 49%. The association between fatty liver and vitamin D deficiency was statistically significant (p = 0.0001) The correlation between vitamin D deficiency and the proportion of patients with fatty liver was found to be statistically significant (p = 0.04). The correlation between the distribution of patients by insulin resistance and the degree of vitamin D deficiency was also statistically significant (p < 0.001) (Table 4).

**Table 1:** Distribution of patients according to clinical characteristics.

Clinical history	Number of patients (n = 100)	Percentage
Abdominal distension	18	18.0
Weakness	12	12.0
Fever	12	12.0
Fatigue	11	11.0
Abdominal distension	7	7.0
Cough	5	5.0
Chest pain	3	3.0
Diarrhea	3	3.0
Dyspnea	4	4.0
Tiredness	4	4.0
Vomiting	4	4.0
Urinary tract infection	3	3.0
Puffiness	3	3.0
Headache	2	2.0
Jaundice	2	2.0
Nausea	2	2.0
Weight gain	2	2.0
Weight loss	2	2.0
Polyuria	1	1.0

**Table 2:** Association between examination findings and serum vitamin D status. ^a^: Chi-square test

Examination finding		Serum vitamin D status			P-value^a^
		Deficiency (%)	Insufficiency (%)	Sufficiency (%)	
Splenomegaly	Present (n = 9)	8 (88.9)	1 (11.1)	0 (0)	0.001
	Absent (n = 91)	37 (40.7)	15 (16.5)	39 (42.9)	
Hepatomegaly	Present (n = 21)	17 (81)	1 (4.8)	3 (14.3)	0.001
	Absent (n = 79)	28 (35.4)	15 (19)	36 (45.6)	
Ascites	Present (n = 7)	7 (100)	0 (0)	0 (0)	0.001
	Absent (n = 93)	38 (40.9)	16 (17.2)	39 (41.9)	

**Table 3:** Association between serum investigations and serum vitamin D status. ^a^: Analysis of variance test

Parameters		Serum vitamin D status			P-value^a^
		Deficiency (Mean ± SD)	Insufficiency (Mean ± SD)	Sufficiency (Mean ± SD)	
Liver function test	Aspartate aminotransferase (IU/L)	34.98 ± 28.49	24.12 ± 7.25	24.31 ± 9.11	0.03
	Alanine transaminase (IU/L)	36.42 ± 34.31	22.44 ± 5.66	25.90 ± 9.26	0.06
Uric acid (mg/dL)		4.24 ± 1.19	4.19 ± 0.98	4.05 ± 1.09	0.73
Platelet count (10^3^/µL)		229.89 ± 121.89	255.44 ± 110.41	274.51 ± 91.16	0.17
Serum total bilirubin		1.25 ± 1.04	0.72 ± 0.36	0.69 ± 0.33	0.002
Serum albumin		3.64 ± 1.01	4.22 ± 0.64	3.99 ± 0.52	0.02
Lipid profile	Cholesterol (mg/dL)	199.36 ± 57.48	39.96 ± 10.72	128.93 ± 42.04	0.01
	High-density lipoprotein) (mg/dL)	158.12 ± 43.63	46.75 ± 11.18	100.38 ± 35.29	0.01
	Low-density lipoprotein (mg/dL)	181.10 ± 37.68	20.21 ± 14.98	108.79 ± 36.37	0.01

**Table 4:** Distribution of patients according to the grades of fatty liver and insulin resistance and its correlation with serum vitamin D status. ^a^: Chi-square test

Variables		Serum vitamin D status			Number of patients (n = 49) (%)	P-value^a^
		Deficiency (%)	Insufficiency (%)	Sufficiency (%)		
Grades of fatty liver	Grade I	10 (62.5)	5 (31.2)	1 (6.2)	16 (32.7)	0.04
	Grade II	9 (75)	1 (8.3)	2 (16.7)	12 (24.5)	
	Grade III	20 (95.2)	1 (4.8)	0 (0)	21 (42.9)	
Insulin resistance	Yes	21 (84)	2 (8)	2 (8)	25 (25)	0.0001
	No	24 (32)	14 (18.7)	37 (49.3)	75 (75)	

## Discussion

Vitamin D deficiency was linked to an increased risk of NAFLD in this cross-sectional research. Although the exact relationship between vitamin D and NAFLD is unknown, several studies have suggested that vitamin D deficiency is linked to the prevalence and severity of NAFLD [13-15]. In adults with normal serum liver enzymes, low vitamin D levels are linked to the development of NAFLD independent of metabolic syndrome. The proportion of female patients was greater than male patients among the 100 patients investigated, with a male-to-female ratio of 36:64. In their research, Lonardo and Suzuki also noted a gender difference [16]. According to a previous study, NAFLD was highly prevalent among women. The gender disparity in the prevalence of NAFLD might be related to differences in fat distribution between men and women. The bulk of NAFLD cases were in their fourth and fifth decades, although there was no significant relationship, according to our data. Vitamin D deficiency was the highest in those aged 41-50 years (54.2%) and lowest in those aged 30-40 years (29.4%). Clinical characteristics were investigated by Basaranoglu and Neuschwander-Tetri, and most occurrences were observed in people in their 40s or 50s, though the entire spectrum has also been reported in children. Obesity and hypertension are common in NAFLD patients. Although many individuals have no symptoms, tiredness and dullness are the most common complaints. Mild-to-moderate hepatomegaly is one of the most prevalent physical examination findings. Patients with NAFLD may have hyperlipidemia, hyperglycemia, hyperinsulinemia, and decreased insulin sensitivity on a biochemical level [17]. Hadizadeh et al. conducted a study and reported analogous findings [18]. The analysis of variance test revealed a significant (p = 0.02) difference in systolic blood pressure between the severity of vitamin D deficiency in our research. The functional crosstalk between NAFLD and hypertension has been reported and discussed by some studies; moreover, in non-hypertensive people, no study has described the salient features of NAFLD. Our findings show that blood pressure is linked to NAFLD in non-hypertensive individuals; both systolic and diastolic blood pressure have been identified as independent risk factors for NAFLD [19]. Patients with NAFLD were shown to have higher body weight, fasting blood glucose, and blood pressure concentration than those without NAFLD (p < 0.05), according to another study. Insulin resistance, which is important in the development of both illnesses, is linked to NAFLD and hypertension. Elevated blood pressure has also been demonstrated as a sign of NAFLD [20]. We also observed that in NAFLD patients, vitamin D deficiency was significantly associated with insulin resistance and higher homeostasis model assessment-estimated insulin resistance values compared to those without insulin resistance. Multiple studies have demonstrated the association between obesity and vitamin D deficiency, with both working synergistically to influence the risk of insulin resistance [21]. Low levels of blood 25(OH)D are inversely linked with markers of obesity, including BMI (30 kg/m2), fat mass, and waist circumference [22]. In bidirectional genetic studies, high BMI was associated with decreased 25(OH)D; each unit increase in BMI was associated with a 1.15% decrease in blood 25(OH)D concentration [23,24]. According to our findings, which are analogous to those of Glass et al. [25], vitamin D deficiency is significantly associated with the presence of hepatomegaly, splenomegaly, and ascites in NAFLD. In our investigation, vitamin D deficiency was also shown to be associated with elevated alanine transaminase and aspartate aminotransferase levels in NAFLD patients. The subsequent results may explain the potential processes underlying the link between vitamin D and NAFLD. Vitamin D influences extraskeletal metabolic organs, which can indirectly influence hepatic lipid metabolism and lower blood TC, TG, and LDL levels, leading to additional liver fat buildup [26]. Vitamin D modulates the immune system and has anti-inflammatory properties. Vitamin D deficiency results in dysfunctional adipose tissue and, consequently, chronic inflammation, which may contribute to the development of NAFLD [27,28]. According to Chatrath et al., NAFLD patients demonstrated atypical lipid metabolism and metabolic syndrome characteristics. NAFLD is characterized by elevated triglycerides, elevated LDL (non-type A), and decreased HDL. NAFLD is caused by the excessive synthesis of very low-density lipoprotein by the liver and the decreased clearance of lipids by the liver, which results in abnormal lipid metabolism. The atherogenic lipid profile of NAFLD is driven by insulin resistance, which may be aggravated by vitamin D deficiency [29].

The distribution of patients according to the grade of fatty liver and its relationship with the degree of vitamin D insufficiency was also discussed in our study. Fatty liver grade, as assessed by ultrasonography, was shown to have a significant (p = 0.0001) relationship with the degree of vitamin D deficiency. In 2014, Park et al. performed a cross-sectional study to investigate the relationship between vitamin D levels and NAFLD and reported a substantial link between vitamin D insufficiency and NAFLD as well [30].

Our study has numerous limitations, including the fact that it was a single-center study based on a small sample, prohibiting generalizations regarding the prevalence of fatty liver in the Indian community. In cross-sectional studies, it might not always be feasible to eliminate selection bias. If patients with complications die prematurely, it can lead to the selection of survivors. This is an imminent problem with older age groups. To establish a stronger link, more research with a larger study population is required.

## Conclusions

Vitamin D deficiency is linked to an increased incidence of NAFLD as well as the severity of NAFLD grade. Estimating vitamin D levels can assist in minimizing the risk of NAFLD. In NAFLD cases, vitamin D supplementation can help slow down the progression of the disease. In this study, vitamin D deficiency was also linked to deranged liver enzymes and dyslipidemia in NAFLD patients.
